# The role of threat sensitivity as a mediator in the relationship between cognitive conflict and risk-taking behavior in young adults

**DOI:** 10.1186/s41235-025-00673-y

**Published:** 2025-09-22

**Authors:** Yashasvi Walia, Rajnish Kumar Gupta

**Affiliations:** Department of Psychology, Manipal University Jaipur, Jaipur, India

**Keywords:** Cognitive conflict, Risk-taking behavior, Threat sensitivity, Mediation, Good health and well-being

## Abstract

Cognitive conflict and risk-taking behaviors are linked in complex ways. This study examined whether threat sensitivity explains the relationship between conflict monitoring and risk-taking in young adults. A sample of 204 university students (ages 18–25, mean = 20.55, SD = 2.14) completed a computerized Stroop task (cognitive conflict), the RT-18 questionnaire (risk-taking), and the TF-44 Trait Fear Scale (dispositional threat sensitivity). Pearson correlations indicated that greater Stroop interference score (higher conflict) was associated with lower self-reported risk-taking and higher threat sensitivity. In turn, higher threat sensitivity predicted reduced risk-taking. Mediation analysis confirmed that cognitive conflict influenced risk-taking indirectly through threat sensitivity. The direct effect of conflict on risk-taking was non-significant, whereas the indirect path via threat sensitivity was significant, suggesting an indirect-only (complete) mediation. These results suggest that individuals experiencing higher internal conflict become more vigilant toward potential threats, which in turn deters them from risky actions. Understanding this pathway could guide interventions (i.e., cognitive control or anxiety-management training) to reduce maladaptive risk behaviors in young adults.

## Significance statement

Young adults often engage in risk-taking behaviors, such as substance use, reckless driving, or unsafe social practices, that pose significant public health and personal risks. While much research has focused on personality traits or peer influence, this study explores a deeper, less understood question: How do internal cognitive processes like conflict monitoring influence risk-related decisions, and what psychological mechanisms mediate this relationship? This research examines the role of threat sensitivity, a biologically rooted tendency to perceive and respond to danger, as a key factor linking cognitive conflict to risk-taking. Using a Stroop task to induce cognitive conflict, along with validated measures of risk-taking and threat sensitivity, the study provides empirical evidence that cognitive conflict indirectly decreases risk behavior by increasing vigilance toward threats. These findings improve our understanding of how cognitive control and emotional regulation together influence real-world decisions. They also have practical implications-interventions that enhance cognitive conflict resolution or modify threat appraisal (e.g., through mindfulness, executive training, or anxiety management) may help reduce harmful risk-taking among youth. The work connects psychological theory with societal needs, contributing to “use-inspired basic research.” It highlights a cognitive-affective mechanism that could be leveraged to promote safer behavior during a critical developmental stage.

## Introduction

Cognitive conflict is a psychological state that arises when an individual’s existing beliefs, ideas, or actions are inconsistent with new information or experiences (Waxer & Morton, 2012). Piaget (1977) argued that such conflict drives cognitive development by prompting individuals to adjust their mental schemas. Contemporary studies suggest that conflict can elicit epistemic emotions (i.e., confusion, curiosity) that deepen task engagement. A prototypical example of such a task is the Stroop color-word task, in which participants must name the ink color of a word while ignoring its semantic meaning. Stroop tasks index the conflict involved in overriding a prepotent response and require continuous monitoring of cognitive control (Scarpina & Tagini, 2017). Prior work showed that Stroop-induced conflict increases sympathetic arousal, anxiety and frustration (Hoshikawa & Yamamoto, 1997). However, the effect of conflict on judgment or performance depends on its intensity; moderate conflict often improves decisions, whereas excessive conflict can impair them. For instance, moderate task conflict can enhance performance, but intense conflict tends to increase stress and hinder outcomes (De Dreu & Weingart, 2003; Parayitam & Dooley, 2011). In educational contexts, eliciting cognitive conflict can foster conceptual change, but if conflict remains unresolved it may block learning (Limón, 2001). Together, these findings indicate that cognitive conflict influences how people adapt their thinking, but its benefits depend on effective conflict management.

Adolescence and young adulthood are periods of uneven brain maturation, with subcortical reward or emotion systems developing faster than prefrontal cognitive control regions (Balogh et al., 2013; Steinberg, 2008). This imbalance contributes to typical increases in risk-taking behavior (e.g., substance use, reckless driving) during these periods. Nonetheless, higher-order cognitive processes still shape how risks are perceived and evaluated (Frederick, 2005). For example, adolescents’ ability to resolve emotional conflict (measured by an emotional Stroop task) predicts their real-world risk-taking better than their ability to resolve non-emotional conflict (Botdorf et al., 2017). Similarly, meta-analysis found that lower general cognitive ability often leads to decision errors resembling risk-taking in experiments (Mechera-Ostrovsky et al., 2022). These observations suggest that risk behaviors can arise from decision-making difficulties or lack of cognitive control as well as from true risk preferences.

A key factor influencing risk-taking is threat sensitivity, defined as the dispositional tendency to perceive potential danger and respond with fear or vigilance (March et al., 2024). This concept is rooted in Gray’s reinforcement sensitivity theory (RST). In RST, the behavioral inhibition system (BIS) regulates responses to conflict and punishment cues by increasing vigilance and behavioral inhibition (Gray & McNaughton, 2003). Under threat, the BIS induces caution, risk aversion, and heightened anxiety, aligning closely with the concept of threat sensitivity. Thus, individuals with a highly sensitive BIS are more prone to perceive and react to threatening cues, which can limit their risk-taking. In young adults, BIS activity has been linked to elevated anxiety, hypervigilance, and internal conflict in risky situations (Corr, 2013). Understanding BIS, therefore, provides a neurobiological framework for interpreting threat sensitivity and its potential mediating role between cognitive conflict and risk behavior.

Although cognitive conflict, risk-taking, and threat sensitivity have been studied extensively individually, their interplay remains unclear. No prior research has directly tested whether threat sensitivity connects these constructs. For example, Schacter et al. (2024) found that social threat sensitivity mediates the association between peer victimization and adolescent anxiety, suggesting a mediating role for threat sensitivity in psychological outcomes. Understanding the role of threat sensitivity in the effect of cognitive conflict on risk behavior is important, as it could reveal mechanisms underlying adaptive versus risky decision-making. The present study thus examined whether threat sensitivity explains the relationship between cognitive conflict (measured by Stroop interference) and risk-taking in young adults. We hypothesized that greater cognitive conflict would be associated with higher threat sensitivity, which in turn would be associated with lower risk-taking. We tested this mediation hypothesis using a simple mediation model. By clarifying this pathway, we aim to inform interventions (i.e., enhancing cognitive control or modulating threat appraisal) to reduce maladaptive risk behaviors in youth.

## Methods

### Sample

The study included 204 participants (118 males and 86 females) recruited from a private university in northwestern India. The choice of sample size was based on simulation studies, suggesting that a sample of approximately 150 would have a desired statistical power of 80% to identify small to medium-sized mediation effects (Newar et al., 2025; Schoemann et al., 2017). Participants included Native Asians, aged 18 to 25 (*mean* ± *S.D.* = *20.55* ± *2.14*). Participation was voluntary and anonymous, and no monetary compensation was provided. The inclusion criteria were an age range of 18–25, an education level of undergraduate or postgraduate, normal or corrected-to-normal hearing and vision, as well as a basic understanding of the English language and computer systems for performing the behavioral assessments. Participants who had a self-reported history of neurological or psychiatric disorders were excluded from the study.

### Measures

#### Stroop task

**Fig. 1:**
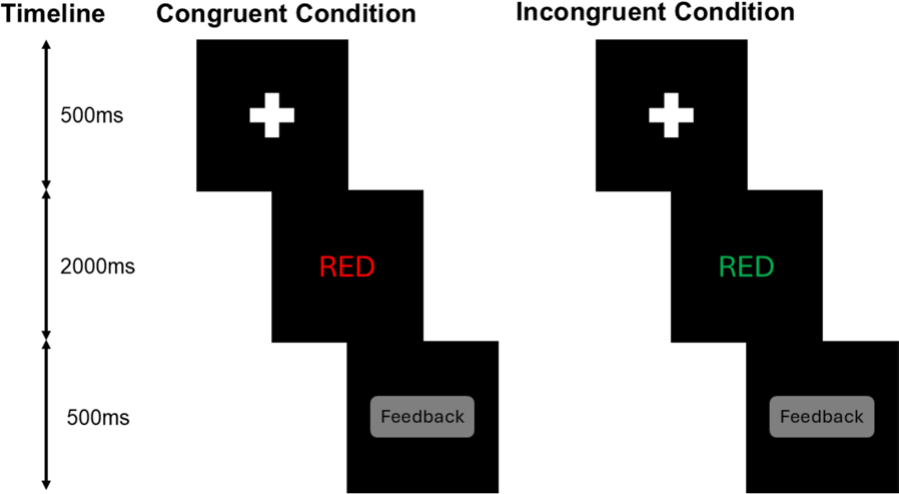
Stroop task. *Note.* The dimensions in the figure are for representational purposes only and are not to scale

Cognitive conflict was assessed using a computerized color-word Stroop task using Psytoolkit (Stoet, 2010, 2017). In line with recent methodological recommendations (Viviani et al., 2024), the task used four color-words (red, green, blue, and yellow), each appearing in four ink colors, with an equal number of congruent and incongruent trials. Trials were randomized to avoid predictable sequences, and practice trials were given to stabilize performance. The stimuli were shown in full-screen mode on a black background at a resolution of 1920 × 1080 pixels. Initially, a fixation stimulus, represented by a plus sign, appeared in the center of the screen for 500 ms. Then task condition stimulus (congruent/ incongruent) was presented until the participant responded or a 2000 ms time-out passed. After each response, feedback was displayed for 500 ms (Fig. 1).

In our study, the Stroop interference score was used as the index of cognitive conflict, calculated as the difference in average response time (RT) between incongruent and congruent trials (Bugg et al., 2008). To get the Stroop interference score, we included only correct trials while excluding the inaccurate and timeout trials in each condition, consistent with the standard practice (MacLeod, 1991). RT on error trials are typically shorter due to speed–accuracy trade-offs and do not reflect genuine conflict processing. Restricting analyses to correct responses ensures that interference effects capture cognitive control demands rather than attentional lapses or guessing. Larger Stroop interference indicated greater processing conflict.

#### RT-18

The RT-18 is a binary response questionnaire with 18 items assessing risk-taking behavior in young adults. It offers characteristic statements (such as "I frequently do things on impulse") that participants can agree or disagree with by responding yes or no. The scale has demonstrated excellent test–retest reliability (*r* = *.94*) and high internal consistency (*Cronbach's alpha* = *.80*–*.89*) (De Haan et al., 2011).

#### Trait fear scale (TF-44)

Dispositional threat sensitivity was measured using the TF-44 Scale (Kramer et al., 2020). It is a 44-item scale that uses a 4-point Likert scale (“True,” “Somewhat True,” “Somewhat False,” and “False”). A higher score on the scale indicates heightened threat sensitivity.

### Procedure

After obtaining informed consent, each participant was invited to the laboratory for a detailed briefing about the study. Administration of all tests was conducted in a quiet and well-lit room. Participants first completed demographic and questionnaire measures (RT-18 and TF-44). Next, the Stroop task was administered on a computer. Following instructions and six practice trials, participants completed 96 trials, which were presented randomly and evenly divided between congruent and incongruent conditions.

### Data analysis

Data were analyzed using the Statistical Package for the Social Sciences (SPSS, version 20). A Pearson correlation was performed to measure the bivariate association between variables. Mediation analyses were performed using the Process Macro v4.2 (Hayes, 2017). We used Model 4 with cognitive conflict (Stroop interference) as the independent variable, risk-taking behavior as the dependent variable, and threat sensitivity as the mediator. Bootstrapping (5000 samples) was used to generate bias-corrected 95% confidence intervals for indirect effects. Statistical significance was set at *p* < .05.

## Result

**Fig. 2:**
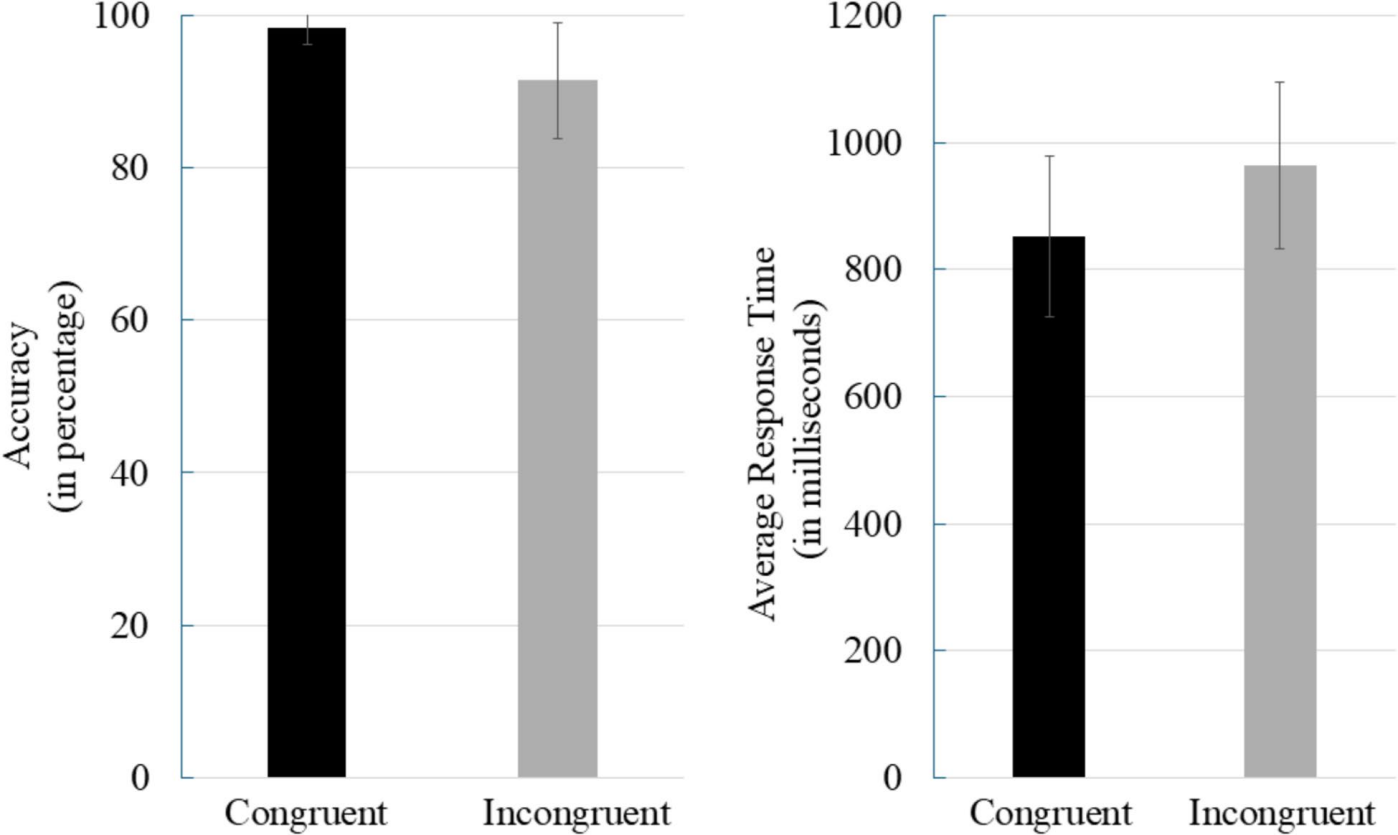
Stroop task performance (*N* = 204). *Note.* Error bars represent standard deviations

Descriptive statistics and correlations are shown in Table 1. To illustrate the Stroop task performance, Fig. 2 shows mean RT (in milliseconds) and accuracy rates (in percentage) for congruent and incongruent trials. As expected, incongruent trials elicited slower RTs and lower accuracy compared to congruent trials. Correlation analysis revealed that greater cognitive conflict (higher Stroop interference) was significantly associated with lower risk-taking (*r* = − .349, *p* < .001) and higher threat sensitivity (*r* = .915, *p* < .001). Additionally, threat sensitivity was negatively correlated with risk-taking (*r* = − .393, *p* < .001). These patterns suggest that participants experiencing more conflict reported higher vigilance toward threats and less inclination to take risks.

**Table 1:** Descriptive statistics and correlation between cognitive conflict, risk-taking behavior, and threat sensitivity (*N* = 204)

Variables	Scores		Cognitive conflict	Risk taking	Threat sensitivity
	mean	S.D			
Cognitive Conflict (in milliseconds)	110.95	46.06		− .349***	.915***
Risk taking	10.75	2.43			− .393***
Threat sensitivity	55.73	20.37			

***p < 0.001

**Fig. 3:**
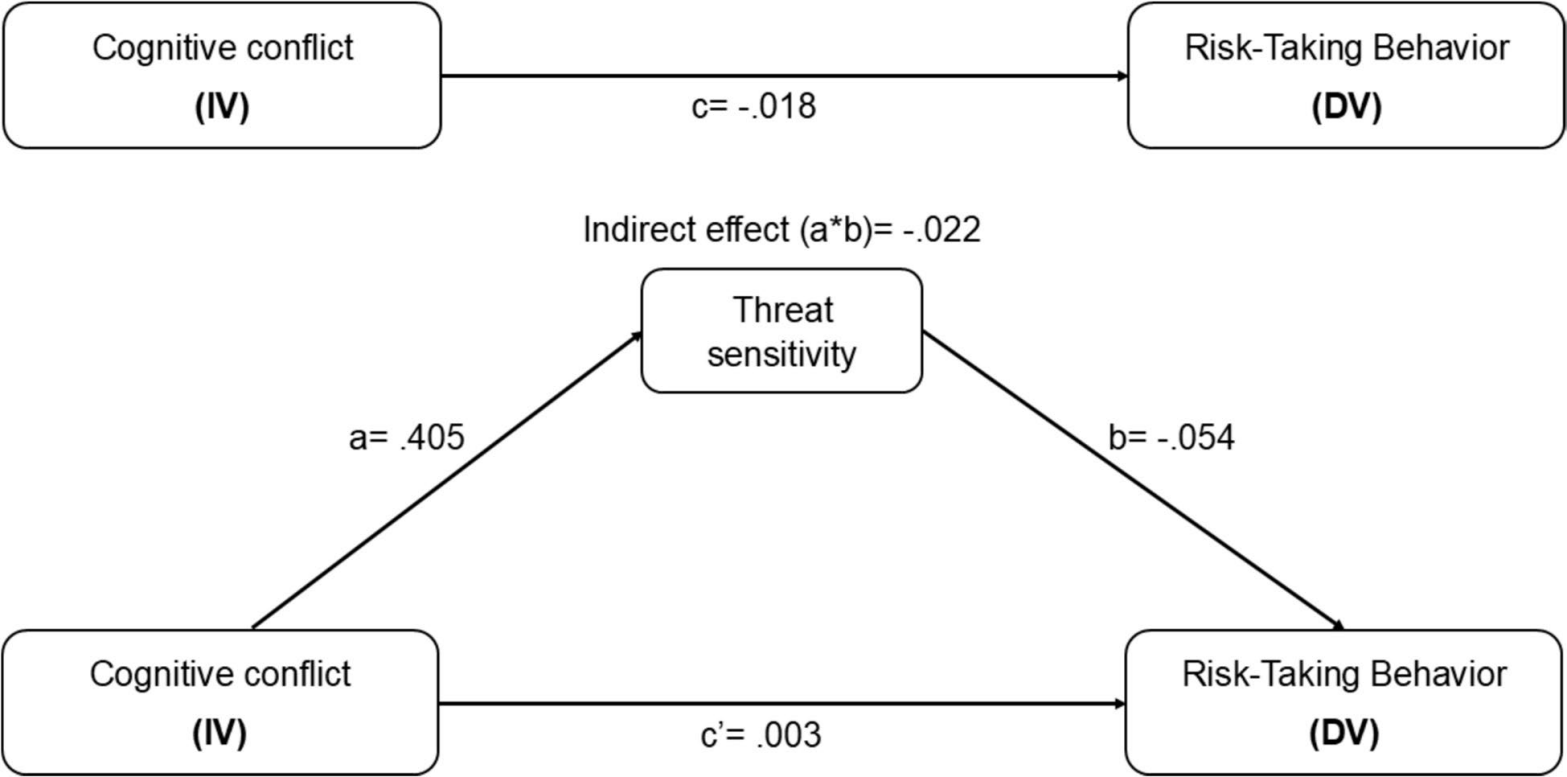
Conceptual mediation model

We then conducted the mediation analysis to determine whether cognitive conflict influences risk-taking behavior indirectly through threat sensitivity (Fig. 3). Mediation analysis indicated that threat sensitivity fully mediated the relationship between cognitive conflict and risk-taking behavior. First, where cognitive conflict significantly predicted threat *sensitivity *(*path a: B* = .405, *t* = 32.336, *p* < .001). Second, when threat sensitivity was included in the model, cognitive conflict no longer significantly predicted risk-taking (*direct effect c′: B* = .003, *t* = .403, *p* = .687). In contrast, threat sensitivity significantly predicted risk-taking (path B: *p* < .001). The indirect effect of cognitive conflict on risk-taking via threat sensitivity was significant (*B* = − .022, 95% CI [− .036, − .007]). Together, these results indicate a complete (indirect-only) mediation where cognitive conflict influenced risk-taking entirely through its effect on threat sensitivity. Table 2 reports the mediation regression coefficients and confidence intervals.

**Table 2:** Mediating analysis between cognitive conflict, risk-taking behavior, and threat sensitivity (*N* = 204)

Effect	Unstandardized Coefficient (B)	Std. Error	*t*-value	*p* value	CI_95_	
					Lower limit	Upper limit
Direct	.003	.009	.403	.687	− .013	.02
Indirect	− .022				− .036	− .007
Total	− .018	.003	− 5.3	< .001	− .025	− .012

*Based on 5000 bootstrap samples

## Discussion

The current study examined the role of cognitive conflict in predicting risk-taking behavior among college students, and whether threat sensitivity mediates this relationship. Consistent with our hypotheses, Stroop-based conflict was inversely related to risk-taking behavior; however, this relationship was indirect, mediated through threat sensitivity. Individuals with higher conflict showed greater threat sensitivity, and those with greater threat sensitivity reported lower risk-taking. In mediation terms, any effect of conflict on risk-taking was fully explained by threat sensitivity, indicating complete mediation (MacKinnon et al., 2007).

Our findings are supported by existing research on cognitive control and risky behavior. Botdorf et al. (2017) reported that adolescents’ risk-taking was predicted by their control over emotional conflict, but not by non-emotional conflict, highlighting that emotional factors mediate the conflict–risk link. Similarly, Romer et al. (2009) found that executive cognitive functions influence risk-taking indirectly through impulsivity rather than exerting a direct effect. These studies imply that neutral conflict resolution alone has a weak direct link to risk behaviors, aligning with our finding that cognitive conflict predicts risk-taking only through an emotional or attentional factor (threat sensitivity). Consistent with this view, Jusyte et al. (2017) reported that convicted offenders experienced less cognitive conflict when breaking rules, indicating a tendency to ignore internal checks on risky behavior. In line with these results, our data showed that lower Stroop interference was associated with higher risk-taking, consistent with the notion that diminished internal conflict facilitates risk engagement.

From a theoretical perspective, our findings align with Reinforcement Sensitivity Theory. According to this theory, a highly sensitive BIS (as measured by our threat-sensitivity scale) heightens conflict monitoring and behavioral inhibition under threat. This, in turn, reduces risky actions. Thus, individuals experiencing more cognitive conflict may become more vigilant about potential dangers, leading them to avoid risks. Recent neurocognitive models suggest that BIS activity interacts with executive control systems to regulate decision-making under uncertainty or punishment (Corr & McNaughton, 2012), a framework supported by our findings. In effect, the path from cognitive conflict to risk behavior in our data was governed not by conflict load alone but by the BIS-mediated emotional reactivity (threat sensitivity) that conflict induced.

The complete mediation suggests two pathways by which cognitive conflict ultimately shapes risk behavior. First, cognitive conflict → threat sensitivity. We observed that participants experiencing greater Stroop interference (more conflict) reported significantly higher threat sensitivity. This may indicate that encountering internal conflict triggers a general state of alertness or uncertainty, making individuals more vigilant toward potential external threats. In other words, dealing with conflicting information might prime the brain’s threat detection systems. Second, threat sensitivity → risk-taking behavior: We also found that higher threat sensitivity was associated with lower risk-taking. This implies that individuals who are more sensitive to danger tend to avoid risky choices. Heightened threat awareness acts as a protective factor, controlling impulsive or hazardous behaviors. These findings aligned with previous work, such as Ristvedt (2024), discussed how individuals who underplay threats (low threat sensitivity) often engage in extreme risk-taking, whereas those high in anxiety avoid risks. Similarly, Ristvedt et al. (2019) found that people with low trait anxiety (i.e., low threat sensitivity) were more likely to neglect medical risks. Laycock et al. (2024) showed that under threat, decision-makers made more erratic, short-term-focused choices, illustrating how threat cues alone can disrupt planning. Our results reinforce that those predisposed to ignore threats are freer to take risks, while sensitive individuals are more cautious.

An important nuance in our results is the pattern of a suppression effect in the mediation. The total (unmediated) effect of cognitive conflict on risk-taking was significantly negative, but when threat sensitivity was included in the model, the direct effect (c′) became slightly positive (though nonsignificant). Such a reversal in effect sign is characteristic of a suppressor-variable process. In mediation terms, suppression occurs when the indirect effect operates in the opposite direction to the total effect, so that including the mediator strengthens or reverses the apparent direct effect. Here, threat sensitivity acted as a suppressor: when it was accounted for, the negative conflict-risk association was essentially “unmasked” through the indirect path, and the direct conflict-risk path was neutralized. This means that the inverse relationship between conflict and risk-taking only manifests when viewed through the pathway of threat sensitivity. Such suppression effects have been documented in mediation analyses (MacKinnon et al., 2000; Rucker et al., 2011) and underscore the importance of examining indirect pathways even when direct effects seem counterintuitive. In our case, these findings highlight that cognitive conflict influences risk-taking predominantly via emotional and attentional processes, rather than through a direct cognitive effect.

It is important to note that conflict does not invariably produce anxiety or negative affect. Some evidence suggests that successfully overcoming conflict can enhance positive emotion and motivation (Schouppe et al., 2015). Thus, conflict may trigger both aversive and rewarding experiences depending on context and coping resources. Our findings highlight one pathway where conflict heightening threat sensitivity, which in turn reduces risk-taking. However, they do not exclude the possibility that in other situations, individuals may cope with anxiety by engaging in risky behaviors (such as drinking alcohol as self-medication). Future work should therefore examine both protective and maladaptive coping responses to conflict-induced affects.

We acknowledge a few limitations in this study. First, all measures were self-reported (except the Stroop), which may introduce response biases. Second, our sample was relatively homogeneous (young university students from one region), limiting generalizability. Third, we used a single task to index cognitive conflict. While widely used, this may not capture all facets of real-world conflict processing. Future research should employ multiple conflict paradigms (e.g., go/no-go or flanker tasks) and sample from diverse populations (including community and clinical samples). Incorporating other potential mediators or moderators (e.g., impulsivity, reward sensitivity) and using longitudinal designs could further clarify how these mechanisms evolve and interact over time.

In summary, our study identifies threat sensitivity as a key mechanism linking cognitive conflict to risk-taking behavior in young adults. When people experience high cognitive conflict, they tend to become more sensitive to potential threats, which in turn suppresses their willingness to take risks. These findings highlight that cognitive challenges influence risk-taking behavior primarily through emotional and motivational pathways. For instance, anxiety and threat-related vigilance engage conflict-monitoring regions i.e., anterior cingulate cortex (ACC) and insula, that bias risky decision-making (Nash et al., 2020, 2021), while approach-motivated individuals may take more risks under anxiety to regulate affect (Leota et al., 2023). Similarly, executive control has been shown to moderate the influence of reward sensitivity on adolescent risk-taking (Kim-Spoon et al., 2016), and reduced error-monitoring signals are linked to higher sensation-seeking and risky choices (Santesso & Segalowitz, 2009). From a practical standpoint, interventions aiming to reduce maladaptive risk-taking could benefit from targeting both cognitive control and threat appraisal. For example, training programs that improve conflict monitoring or instruct strategies for managing anxiety may help at-risk youth make safer decisions. In educational and clinical contexts, assessing individuals’ threat sensitivity could inform personalized support, those with low threat sensitivity might benefit from heightened awareness of consequences, whereas highly sensitive individuals might need help coping with anxiety and uncertainty. Overall, integrating cognitive control and emotional appraisal perspectives may yield the most effective approaches to promoting adaptive decision-making in adolescents and young adults.

## Data Availability

The datasets generated during and/or analyzed during the current study are available from the corresponding author on a reasonable request.
